# i-ECO: a novel method for the analysis and visualization of fMRI results in Psychiatry

**DOI:** 10.1192/j.eurpsy.2022.554

**Published:** 2022-09-01

**Authors:** L. Tarchi, T. Fantoni, T. Pisano, S. Damiani, P. La Torraca Vittori, S. Marini, N. Nazzicari, G. Castellini, P. Politi, V. Ricca

**Affiliations:** 1 University of Florence, Department Of Neuropsychiatric Sciences, Florence, Italy; 2 AOU Meyer, Neurology Unit And Laboratories, Florence, Italy; 3 University of Pavia, Department Of Brain And Behavioral Sciences, Pavia, Italy; 4 University of Florida, Department Of Epidemiology, Gainesville, Florida, United States of America; 5 CREA, Research Centre For Fodder Crops And Dairy Productions, Lodi, Italy

**Keywords:** ReHo, Eigenvector Centrality, fMRI, fALFF

## Abstract

**Introduction:**

The high technical barrier to entry in the field of neuroimaging can hinder early insight from promising results and the development of evidence-based clinical practice.

**Objectives:**

The working group focused on published literature in order to develop a new methodology in the analysis, visualization, and representation of fMRI data in the psychiatric setting.

**Methods:**

Three valid and established measures were chosen, in order to achieve dimensionality reduction, stability and explainability of results, namely Regional-Homogeneity; fractional Amplitude of Low-Frequency Fluctuations; Eigenvector-Centrality. Each measure was color coded and individual images per subject compiled, averaging results by functional networks as described the FIND lab of the University of Stanford. 272 individual scans were processed (130 neurotypicals, 50 patients with Schizophrenia, 49 with Bipolar Disorder, 43 with ADHD).

**Results:**

The discriminative power between clinical groups of the novel method was significant both by human eye, and later confirmation by statistical tests, and by computer vision algorithms (Convolutional Neural Networks). The precision-recall Area Under the Curve, dividing by 80/20 proportion between train and test sets, was >84.5% for each group. The group of patients with Bipolar Disorder showed a partial overlap with the group of patients suffering from Schizophrenia – by a dominance of Eigenvector-Centrality and Regional-Homogeneity, as well as a lower prevalence of fractional Amplitude of Low-Frequency Fluctuations, for both in comparison to controls.

**Conclusions:**

The present study offers preliminary evidence for the adoption of i-ECO (integrated-Explainability through Color Coding) in fMRI analyses during rest in the Psychiatric field.

**Disclosure:**

No significant relationships.

